# Social environment affects vocal individuality in a non-learning species

**DOI:** 10.1038/s41598-025-29387-3

**Published:** 2025-12-15

**Authors:** Malavika Madhavan, Lucie Hornátová, Martin Šálek, Alexandra Průchová, Pavel Linhart

**Affiliations:** 1 Department of Zoology, Faculty of Science, University of South Bohemia, České Budějovice, Czech Republic; 2 Institute of Vertebrate Biology, Czech Academy of Sciences, Brno, Czech Republic; 3 Faculty of Environmental Sciences, Czech University of Life Sciences Prague, Prague-Suchdol, Czech Republic; 4 Center for Sustainable Landscapes under Global Change (SustainScapes), Department of Biology, Aarhus University, Aarhus, Denmark

**Keywords:** Acoustic niche, Bioacoustics, Owl, Population density, Signal evolution, Vocal learning, Vocal plasticity, Ecology, Ecology, Evolution, Zoology

## Abstract

Individual recognition is fundamental to the social behaviour of many animals. In the context of territorial behaviour, animals in high-density populations encounter conspecific rivals and potential mates more frequently, which should enhance the individuality of territorial signals to facilitate recognition among conspecifics. We investigated vocal individuality in male territorial calls of two populations of little owls (*Athene noctua*) with different densities. Further, to explore the potential influence of local population distribution on individuality, we also examined isolated males without neighbours and clumped males with neighbours. Our findings indicate higher individuality at higher densities across both scenarios, measured using two individuality metrics: Beecher’s information statistic and Discrimination score. Clumped males exhibited significantly lower acoustic niche overlaps (i.e. higher vocal individuality) compared to isolated males. However, only a non-significant trend for lower acoustic niche overlaps (i.e. higher vocal individuality) was found for males from high density compared to low density populations. This suggests that the immediate social environment might be more influential than larger-scale population density patterns. This study suggests that vocal individuality in a territorial species is influenced by conspecific density, similar to findings in group-living and colonial species.

**Supplementary Information:**

The online version contains supplementary material available at 10.1038/s41598-025-29387-3.

## Introduction

Population density can independently affect both the fitness and behaviour of animals. For example, population density may affect levels of intraspecific competition^1–4^, spatial behaviour such as dispersal^5^ and home range size and use^6,7^. High population densities may compromise fitness through declines in reproductive parameters caused by competition^8^ or predation^9^. Even mating systems can be shaped by the density of a population^10,11^.

Population density can also shape how individuals communicate with each other^12^. One of the communication tasks that might be likely affected by population density is individual recognition. Large pools of individuals and repeated interactions occurring between them may demand adaptations in signal-design in the process of individual recognition. For a signaller to efficiently and reliably signal their identity information, between-individual variation should consistently be much greater than within-individual variation of a signal^13,14^. Group-living animals represent a specific case of increased population density clusters. Fitting with this, animals living in larger and more complex social groups should have more complex communicative signals as suggested by the social-complexity hypothesis^15,16^; reviewed in Peckre et al.^17^. Strong individual signatures have been found in acoustic, visual and olfactory signals of many group-living and colonial species^18–22^. Studies on colonial and non-colonially breeding swallow species showed the particular importance of acoustic signal-design for individual parent-offspring recognition in species living in high population densities, where calls of colonial species were longer and more frequency modulated, leading to about 18 times more individual signatures in their calls than in non-colonial species, allowing for better discrimination^23–25^. Similarly, Martin et al.^26^ found that local population density affects individuality in mother-pup attraction calls in Cape fur seals (*Arctocephalus pusillus pusillus*). Several other studies report that species living in larger groups/colonies display larger individuality in their calls^27,28^.

However, strong individual signatures are also frequently found in solitary-living, territorial species. In such a territorial context, it is well established that individuals need to tell-apart neighbours from strangers and also differentiate among neighbours - the ‘dear-enemy effect’^29–33^ to manage and minimize unnecessary territorial aggressive interactions^34^. However, drivers of high vocal individuality in territorial species were mostly neglected. None of the few previous studies revealed an effect of population density on vocal individuality in a territorial species^35,36^, which is somewhat interesting as territorial species should benefit from higher distinctiveness in denser populations to remain well recognizable for their neighbours among larger pool of potential competitors.

Acoustic signals are an ideal system to study identity signals because they can be used for immediate long-range communication. Further, acoustic communication can be highly important for many nocturnal species. Indeed, acoustic communication might have evolved to facilitate communication in reduced light conditions^37^. Nocturnal animals prioritise vocalisations over other channels of communication^38,39^. Also, due to their dynamic and multidimensional nature, acoustic signals allow substantial scope for modifying signals by changing their spectral and/or temporal qualities. Therefore, nocturnal birds such as owls, are excellent models for studying acoustic communication. Owls are known to have individually distinct calls^40–42^, see reviewed in Madhavan & Linhart^43^, which they use to discriminate among one-another for purposes such as advertisement and territorial defence^44,45^, mate-recognition^46^, or parent-offspring recognition^46,47^.

The little owl (*Athene noctua*) is a small-sized (body mass = 170–210 g), territorial (high fidelity to occupied territories^48^), owl species with a large natural distribution range, stretching across much of Eurasia and North Africa, where it inhabits a variety of open landscapes which include semi-arid grassland steppe, rocky outcrops, and woodland edges^48–50^. Little owl population densities steeply declined across different parts of its distributional range in recent decades^48^, with the most profound population declines in Central and Western Europe^49,51,52^, where they are currently most commonly associated with human-dominated agricultural landscapes, with grasslands, croplands, orchards and gardens representing crucial foraging areas^48,50^. However, in areas with favourable habitat conditions such as traditionally managed small-scale farmland with semi- natural grasslands^53,54^, and traditional human settlements (villages) with old buildings providing many suitable nesting sites in holes, crevices, or roof cavities^49,55^, little owls can still thrive. Thus, little owls can be found in a wide range of population densities ranging from very low (e.g., 0.009–0.033 calling males/km^2^^49,56^), to very high (e.g., 20–33 calling males/km^2^^57,58^), with nearest neighbours being tens of metres to several kilometres apart from each other. Little owl males produce territorial ‘*hooook*’ calls that they use to defend their territories^48^. In ideal conditions, territorial calls of little owls can be heard up to 4.4 km^59^, but realistically, due to various environmental conditions such as habitat structure in individual human settlements, wind, rain and mist, most calls can be detected within a 200 m radius of the focal male^60^. Male territorial calls have been shown to be individually vocally distinct^40^, and males can also distinguish between the territorial calls of familiar neighbours and unfamiliar strangers^61^. Further, little owls are a relatively long-lived and sedentary species, both of which contribute to stable relationships between territorial neighbours and increase the potential benefits of individual recognition (e.g. ^62^).

**Fig. 1:**
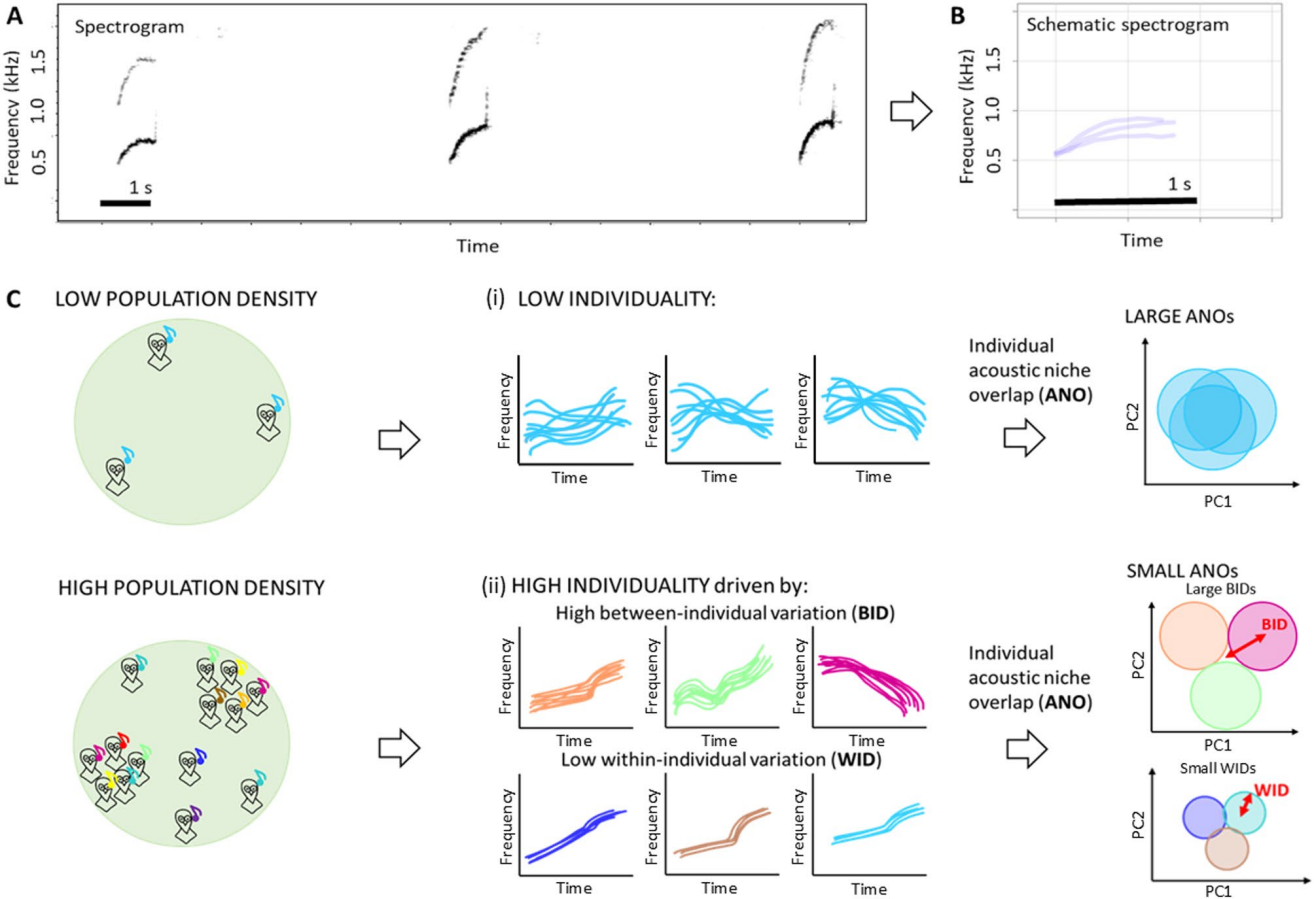
Little owl calls and overview of predictions. (**A**) a spectrogram showing three territorial calls of a single little owl, (**B**) a schematic representation of the same three calls shown in A, plotted on top of each other, made using duration and peak frequency contour measurements of the calls, and (**C**) a schematic representation of our predictions, showing (i) low individuality represented by large acoustic niche overlaps between individuals living in low population densities, and (ii) high individuality represented by small overlaps between individuals living in high population densities, driven by either high between-individual variation, and/or low within-individual variation. Each schematic spectrogram in (**C**) represents a different individual, and similar to (**B**), each coloured line represents call-contours of individual calls, made using peak-frequency and duration of the calls.

Our aim in this study was to compare the territorial calls of little owls from populations with low and high density to see if population density might drive the level of vocal individuality in a species that does not live in groups or colonies. We measured the acoustic features of little owls’ calls and used them to calculate different metrics of vocal individuality. We compared vocal individuality in two spatially separated populations with different average densities (i.e., low- and high-density population). It should be noted that due to logistical constraints we could only compare vocal individuality in two different populations. More replicates from different populations would be needed to establish causal relationships between population density and vocal individuality. To mitigate this issue, we capitalised on the spatial distribution of the high-density population, and we further compared vocal individuality in isolated males (isolated farms, with closest neighbours being several hundreds of metres apart), and clumped males living in dense aggregations of conspecifics (villages, with one to several neighbours in adjacent territories). While macroscopic differences in vocal individuality between the two populations could be explained by longer-term adaptations and genetic differences between the two populations, local differences at the level of a single population might rather suggest a plastic developmental response to current environmental conditions. We predicted the following (Fig. 1):

- Individuals living in the high-density population have higher vocal individuality than those living in the low-density population (between population comparison).
- Clumped individuals with neighbours will have higher individuality than isolated males (within population comparison).

Individuality has two different components: how much signals differ between individuals (between-individual variation) and how consistent they are within each individual (within-individual variation)^13,14,63^. Increase in individuality can be mechanistically driven by an additional increase in between-individual variation or by reducing within-individual variation, or both. Therefore, we characterised each of these individuality components separately and compared their values depending on population density, to explain the source of any possible change in individuality related to population density.

## Methods

### Study sites and recordings

Little owl males were recorded between March and April in both 2013 and 2014. This period overlapped with the courtship period, when little owl males most actively produce territorial hoots^64^. The research was conducted in two study sites: the first one in Northern Bohemia, Czech Republic (hereafter ´LOW density population´) (GPS: 50.41°N, 14.04°E), covering an area of 1493 km^2^ and characterised by intensively managed agricultural hayfields and cropfields^50^. In contrast, the second study site close to Hortobágy National Park, Hungary (hereafter ´HIGH density population´) (GPS: 47.57°N, 20.93°E), covering an area of 1555 km^2^ is characterised by villages surrounded by *Puszta* - the largest contiguous European grasslands - and farmland. The *Puszta* is a landscape mosaic formed by large floodplains, alkali grassland, salt steppe, and arable land^65^, which offer rich foraging resources for little owls^48,53^. Within the villages, non-modernized terraced houses, farmsteads and gardens with domestic animals (poultry, sheep, horses) along with small-scale farming, fruit orchards are typically found features within little owl territories^49,58^. The average little owl density in the LOW-density population was 0.09 calling males per 10 km^2^^56^, and in the HIGH-density population, the average density was 5.01 calling males per 10 km^2^, which is one of the highest reported densities in central Europe^57^. However, within the human settlements of the Hungarian population, densities of little owls are even higher and can reach up to 33.3 calling males per km^2^^57,58^, males therefore frequently have one or several calling neighbours within a 200 m radius (hereafter ´CLUMPED males´ - high local population density). On the other hand, isolated males without any detected neighbours were found on isolated farms in the same population^57^. The closest human settlements to these isolated farms, which might provide suitable breeding habitats for little owls, were 552–1445 m away^66^. The farms, being surrounded by farmland or grassland had no other nesting opportunities for little owls (hereafter ´ISOLATED males´ - low local population density), as breeding sites of little owls are currently exclusively located in human-made structures^49,57,67^. Presence of neighbours was noted during acoustic surveys conducted in the same season when recordings were made, and confirmed by scanning the recordings for territorial calls of the focal male and any other males. We followed the same survey methods as in Šálek et al.^57^, (for more details on the survey protocol, see Supplementary file, S4).

**Fig. 2:**
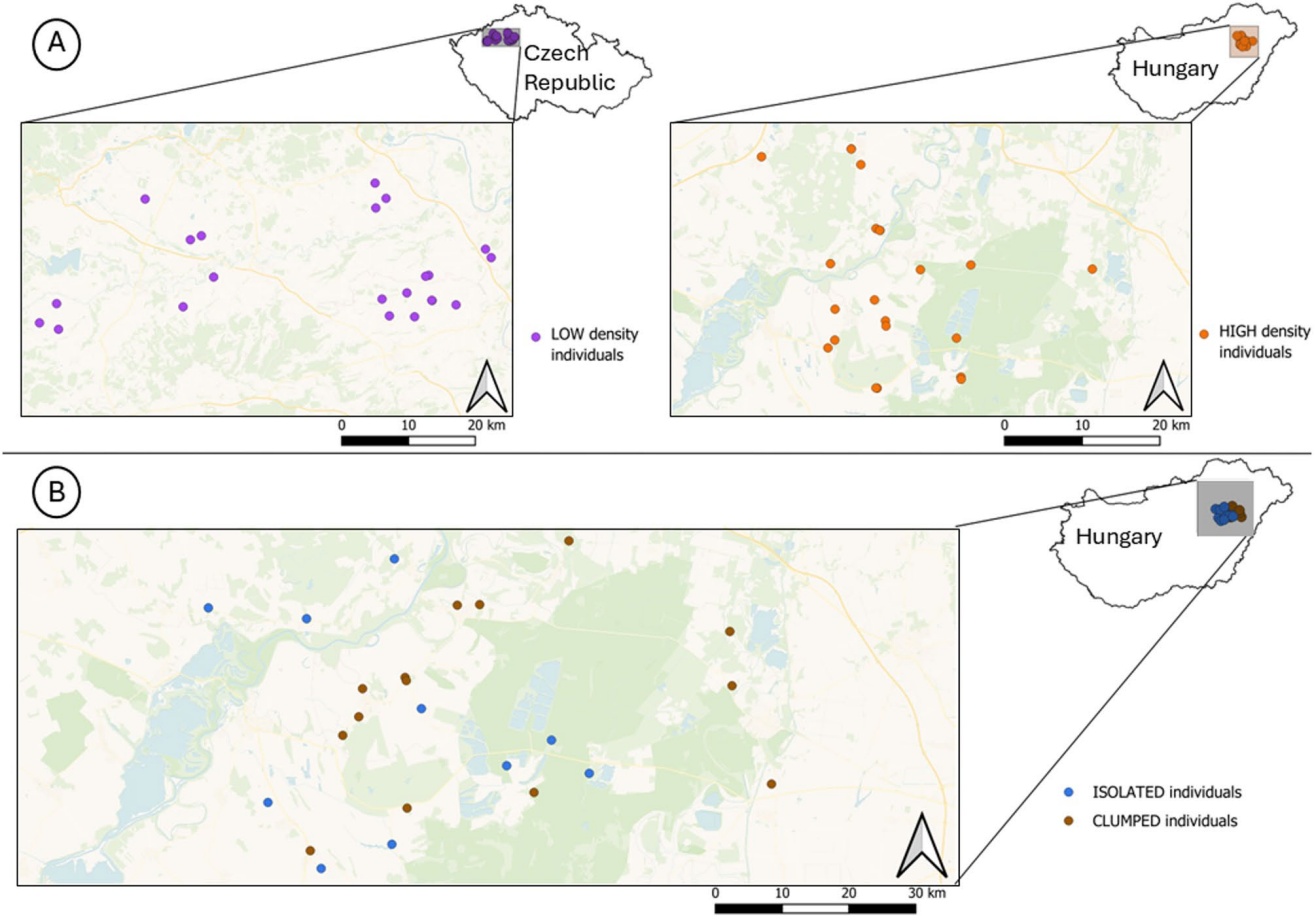
(**A**) Between-population comparison - a map of the study areas in the Czech Republic and Hungary showing distribution of sampled males in LOW-density and HIGH-density populations respectively. (**B**) Within-population comparison - map of the study area in Hungary showing the distribution of ISOLATED and CLUMPED males. Map was created by the authors in QGIS 3.30 (http://qgis.org*).*

Territorial calls of male little owls were recorded in optimal weather conditions (no strong winds or rain), and during the peak of calling activity (between dusk and midnight). For comparison between populations (HIGH vs. LOW-density populations), provoked recordings were made with a Marantz PMD660 solid-state recorder (sampling frequency: 44,100 Hz, 16-bit, WAV format without any compression) and a Sennheiser ME67 directional microphone with Rycote windshield protection. All males were recorded after ca. 1-minute-long playback provocation, from up to 50 m from the focal individual. For the comparison within the HIGH-density population (CLUMPED vs. ISOLATED males) passively recorded spontaneous calls without playback provocation were used. Recordings were made using autonomous recorders Olympus DM-650 programmed to record continuously over night from 7 PM to 7 AM (sampling frequency: 44100 Hz, 16-bit, WMA format). Recorders were positioned within 50 m from the preferred calling posts identified during the previous population census with the use of playback. Often, recorders operated on the localities over two consecutive nights. Nights containing more hooting sequences of better quality were selected for further analysis. Calls from the autonomous recorders were carefully examined to ensure that only non-overlapping calls from the focal male were selected for further analyses. We did this by selecting continuous call sequences from the focal male, who was defined as the closest male calling in the recordings i.e. the signal with the highest amplitude (see Supplementary information, Figure S1). Overall, 32 unique males were recorded in 2013, (13 in LOW; 11 in HIGH; 6 in CLUMPED, 5 in ISOLATED) and 31 unique males were recorded in 2014 (9 in LOW; 11 in HIGH; 8 in CLUMPED; 5 in ISOLATED). Sometimes, provoked calls of males from the HIGH-density population were matched by recordings of spontaneous calls of the same males in the ISOLATED (1 male) and CLUMPED (4 males) conditions. Maps of the study areas indicating individuals from different density conditions are presented in Fig. 2 (note: map does not represent total population density, it only shows recorded individuals). Two types of recording protocols (provoked and spontaneous) were originally aimed for different purposes. Spontaneous recordings were originally collected for analysis of vocal activity in clumped and isolated males^66^ while provoked recordings were collected rather opportunistically for general analysis of individuality in calls^40^. Because different sites were visited each year to make the recordings, and because of the high site fidelity and long-term territories of little owls^48,68^, no male was recorded in both years, and very few males were recorded with both playback and spontaneous methods (see Supplementary information, Figure S1).

### Processing of acoustic data

All processing of recordings, and measurements were done in RavenPro version 1.6^69^. As the fundamental frequency of little owl territorial calls was always between 500 Hz and 2000 Hz (mean ± standard deviation of minimum frequency = 632 ± 117; maximum frequency = 1003 ± 109), we band pass filtered recordings (500–2000 Hz) and reduced the sampling frequency to 4000 Hz before analyses to get better frequency resolution in the spectrograms. Spectrograms of all recordings were produced using the following settings: window size = 512 samples, with Hann type window, window overlap = 93.75% (temporal resolution = 8 ms; spectral resolution = 7.81 Hz).

In the first analysis (comparing individuality in HIGH- and LOW-density populations), we used recordings obtained from 22 territorial males for each population (29 ± 9 calls per male in LOW and 26 ± 9 calls per male in HIGH population). In the second analysis (comparing individuality in CLUMPED and ISOLATED males), we used spontaneously recorded calls from 24 males. This consisted of 14 CLUMPED males and 10 ISOLATED males. A mean of 25 ± 1 calls per male from CLUMPED males, and 24 ± 1 calls per male from ISOLATED males, with minimal background noise from each male were selected and measured from sequences after evaluating for their quality (no overlap with background noise, wind, etc.).

For both analyses, we measured the duration and frequency modulation (Peak Frequency Contour - PFC in RavenPro) of calls. Frequency modulation is very prominent in little owl male calls. It has been shown to be individually distinct and perform better for individual identification than using spectral features or spectrogram cross-correlation^40^. PFC measurements were visualised and manually checked. Calls in which the contour was not properly traced were omitted from further analyses. As RavenPro takes PFC values for each spectrogram slice of the call, the number of PFC values varies depending on the duration of the call, however, further analyses required the same number of measures for each call. To overcome this problem, we used the R package *Rraven*^70^ to conduct dynamic time warping on the calls, using the function ‘*extract_ts*’. This allowed us to extract 10 PFC measurements for each call, which are spread out evenly through the call even when call durations differed. Previously, 10 sampling points were found to be adequately representative of the PFC for individual identification^40^.

### Statistical analyses

#### Quantification of vocal individuality

We used different metrics to assess individuality in little owl territorial calls. Beecher’s statistic (HS), and Discrimination score (DS) are two common sample-wide individuality metrics (i.e. a single individuality value is obtained for the whole population of individuals). DS has been used widely in different studies, but HS is better for comparison between studies with different sample sizes – both of individuals and calls^13,71^. HS and DS were both calculated using the R package *IDmeasurer*^71^. For statistical assessment of individuality, we used metrics obtained at the level of each individual: average within-individual variation and average between-individual variation were assessed to investigate changes in each of the individuality components separately, and an aggregate acoustic niche overlap was calculated as a metric integrating both components together.

**Beecher’s information statistic (HS)** is a method rooted in information theory, which quantifies how well a trait can function as a signal of individual identity^13^. It is calculated using the ratio of total variation (total sum of squares) of the specific condition (HIGH, LOW, CLUMPED, ISOLATED) to within-individual variation (within-sample sum of squares). It lies on a scale from zero to infinity, with zero meaning no individuality. The higher the value of HS, the easier it is to discriminate between individuals. HS has important advantages over the other metrics^71^ and can serve as a comparative metric across species^71,25^. It has been used to calculate individuality in several studies, across species and sensory modalities^27,40,72,73^. As HS requires the input trait variables to be uncorrelated, we first centred and scaled all variables (10 PFC values and duration) and then performed a Principal Components Analysis (PCA) on them using the function ‘calcPCA’ (package *IDmeasurer*). The function ‘calcHS’ from the same package calculates HS for each principal component and then sums them through the whole call to compute the overall Beecher’s statistic HS of the signal. The PCA plots can be found in the Supplementary information (Figures S2 – S3).

**Discrimination score (DS)** is the accuracy score of calls being correctly classified to an individual and is expressed as the percentage of correctly classified calls. We used the function ‘calcDS’ (package *IDmeasurer*), which uses Linear Discriminant Analysis (LDA) with leave-one-out cross-validation to compute the DS for the whole pool of individuals. LDA has been widely used in studies reporting vocal individuality^74–76^. However, it is more susceptible to estimation biases caused by the number of included individuals and calls per individual. To prevent these biases, we aimed to select similar numbers of individuals and calls for our comparisons.

**Within and between individual variation (WID, BID)**. To be an efficient means of recognition at the individual level, the between-individual variation of an identity trait must be consistently greater than the within-individual variation of the same trait^13,14^. We used the same PCA scores from the previous analysis used to compute HS (see above) and Euclidean distances among the calls to characterise within and between-individual variation. To assess within-individual variation, we measured the Euclidean distances from each call of an individual to that individual’s centre point (PCA values averaged over the calls from an individual). These measurements were averaged to obtain a mean within-individual acoustic distance (WID) for each male. High individual call consistency would be characterised with low WID values. To assess between-individual variation, we avoided measuring pair-wise distances between individuals. Instead, we used an approach similar to that used for calculating WIDs. We measured the Euclidean distances from the centre value of each population (PCA values averaged over all calls from the population) to the centre value of each individual of that population (PCA values averaged over the calls of each single male). This male’s acoustic distance from the population center was used as an indicator of between-individual distances (BID) similar to between-group variance in an F-test. Larger BID value would indicate males spread on larger acoustic space, and hence, males with larger pairwise acoustic distances among them.

**Acoustic niche overlap (ANO)**. Niche theory emerged as describing each species in an ecosystem to have an ecological niche which occupies ‘n-dimensional hypervolumes’^77^. Ecological niches and their overlaps are typically used to study resource partitioning between different species in an ecosystem. Since then, there have been developments in how niches are calculated as well as its applications in different branches of ecology^78–80^. In animal communication, the concept of ecological niche has been applied to studying separation of acoustic space between species^81–83^. A similar logic can be also applied to the partitioning of acoustic space among different individuals belonging to the same species. We assume that high individuality results in low overlaps of acoustic features between different individuals, enabling reliable discrimination among individuals. The R package *‘nicheROVER’*^84^ was designed to calculate multidimensional ecological niche region sizes and the overlaps of those niches; we adapted this approach to calculate individual acoustic niches. The package ‘nicheROVER’ (function: ‘overlap’) finds the probability of any call produced by the focal individual being within the acoustic niche of another individual. We calculated all pairwise niche overlaps within each sample of individuals (alpha = 99%, number of Monte Carlo draws = 10,000). Then, we calculated the final aggregate acoustic niche overlap metrics for each individual by summing the probabilities of that individual being in the acoustic niche of any other individual of its population.

#### Comparing populations and subpopulations

HS and DS values could not be statistically compared as they represent population-wide metrics. We used non-parametric Wilcoxon’s rank sum tests (R package *‘stats’* version 4.2.0) to compare WIDs, BIDs, and ANOs between the different population density conditions (HIGH vs. LOW and CLUMPED vs. ISOLATED). The number of males in different statistical tests was always the same (HIGH: *n* = 22; LOW: *n* = 22; CLUMPED: *n* = 14; ISOLATED: *n* = 10). All statistical analyses were carried out in R version 4.2.0^85^. We used the value of α = 0.05 as a threshold for significance of the results.

#### Ethical note

Recordings were made in places with unrestricted public access and on wild animals. This study was purely observational and minimally invasive, except for using playback provocation prior to making some of the recordings; therefore, no special permits were required. We acknowledge the impacts that playback provocation may have on various behavioural aspects of wild animals^86–88^.

## Results

### Individuality in high- and low-density populations

**Fig. 3:**
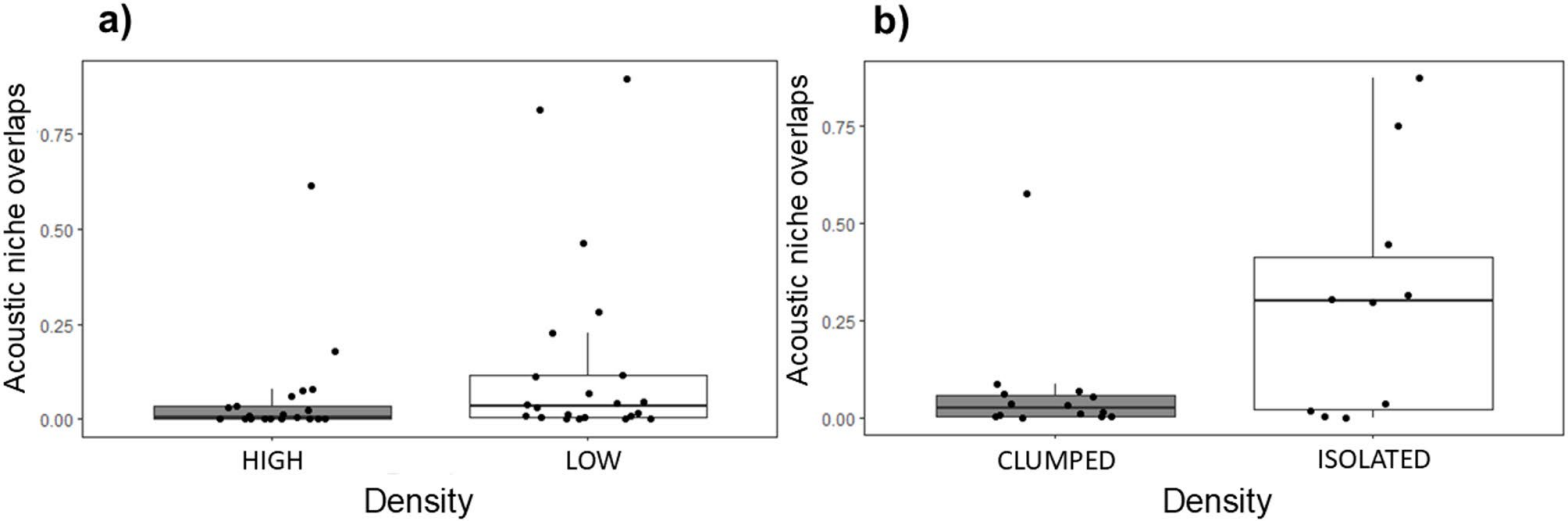
Boxplot with differences in aggregate ANO values **(a)** Between populations; HIGH density and Low (LOW density) and **(b)** Within population; High (CLUMPED males with neighbours) and Low (ISOLATED males without neighbours). Medians, quartiles and non-outlier minima and maxima, along with original data points (dots) are displayed.

We calculated Beecher’s statistic and discrimination score, and both metrics indicated higher individuality in the HIGH-density population (HS = 6.38, 83 unique signatures; DS = 87.7%, 22 individuals, 26 ± 9 calls per individual) compared to the LOW-density population (HS = 4.96, 31 unique signatures; DS = 79.4%, 22 individuals, 29 ± 9 calls per individual). We did not find a significant difference in either WID values (W = 198; *p* = 0.31; HIGH density, median = 0.88, Inter-quartile range (IQR) = 0.42; LOW density,median = 1.12,* IQR = 0.84)* or BID values (W = 262; *p* = 0.65; HIGH density, median = 2.81,* IQR = 1.22; LOW density*, median = 2.41,* IQR = 2.08*) between both populations. Despite not seeing significant differences between the two groups, we still observe that the median values for both comparisons are in the direction of higher individuality in the HIGH-density population. Similarly, when comparing the ANOs of male calls in HIGH and LOW density populations, we did not find a significant difference, but observed that ANOs in the HIGH density population tended towards being lower than those in the LOW density population (W = 165, *p* = 0.07; HIGH density, median = 0.0059,* IQR = 0.05; LOW density*,median = 0.0357,* IQR = 0.16;* Fig. 3a).

### Individuality in CLUMPED and ISOLATED males

As in the first analysis, both Beecher’s statistic and discrimination score indicated higher individuality in CLUMPED males (HS = 5.29, 39 unique signatures; DS = 87.5%, 14 individuals, 25 calls per individual) when compared to ISOLATED males without neighbours (HS = 3.98, 15 unique signatures; DS = 78.9%, 10 individuals, 25 calls per individual). Here again, we did not find a significant difference in either WID values (W = 61; *p* = 0.63; CLUMPED males, median = 1.31,* IQR = 0.47; ISOLATED males*,* median = 1.22*,* IQR = 1.03)* or BID values (W = 81; *p* = 0.55; CLUMPED males,* median = 2.35*,* IQR = 1.45; ISOLATED males*,* median = 2.32*,* IQR = 0.56*) between the two density conditions. However, aggregate ANOs in CLUMPED males was significantly lower than those of ISOLATED males (*W = 33*, *p* = 0.04; CLUMPED males,* median = 0.0223*,* IQR = 0.05; ISOLATED males*,* median = 0.2997*,* IQR = 0.39;* Fig. 3b), suggesting a higher level of individuality in CLUMPED males.

## Discussion

Here, for the first time, we document that some measures of vocal individuality are associated with the density of conspecifics in a species that does not live in social groups or colonies. This is supported by two population-wide individuality metrics. Further, we used additional metrics that allow statistical comparisons of individuality between populations. We did not observe significant differences in individual acoustic niche overlaps between the two populations with contrasting densities. However, within the high-density population, local aggregation of males (CLUMPED males) had significantly smaller acoustic niche overlaps (i.e., higher individuality) than ISOLATED males. When investigating potential mechanisms leading towards higher vocal individuality in CLUMPED males, we did not find significant support for CLUMPED males making their calls more distinct from other males (larger between individual distances, BID), or for them calling in a more consistent way (smaller within individual distances, WID). Higher individuality in CLUMPED males thus seems to be a result of a smaller cumulative combination of both mechanisms.

Our study brings another example on how population density can affect acoustic signals. High conspecific density has been previously shown to upregulate various vocal parameters, notably song rate^89–91^, but also duration^92^, dawn-song onset^93^, and song-type switching^94^ among others (for a review on this topic, see Sánchez & Mennill^95^,. Further, our findings are broadly consistent with the social complexity hypothesis^15^, where increased complexity in acoustic signalling - including individuality, have been linked with increasing social complexity across many species (see^17^, for a detailed account of such examples). High population density is considered one among the different proposed indicators of increasing social complexity. However, studies dealing with the social complexity hypothesis mostly focus on species’ vocal repertoire or song parameters rather than individually distinct vocalisations of the same call-type. A major driver of individuality is to be recognizable among a large pool of individuals. Individuality could be selected for in large groups to avoid unnecessary conflict, facilitate easy recognition in a crowd, or to maintain, or increase genetic diversity^96,97^. The few studies that address communication complexity in individually specific calls, typically study these mechanisms at an interspecific level (but see^26^), and in group-living or colonial species^18,20,98^. Higher individuality has been found in animal species living in larger or more complex groups^27,28,99^. Perhaps one of the best examples so far of individuality being selected for at high densities is shown in the comparative study of four swallow species^23^. Chicks of Sand martin (*Riparia riparia*) and Northern Rough-winged swallow (*Stelgidopteryx serripennis*), which are colonial breeders, show much higher vocal individuality than those of the closely related Barn swallows (*Hirundo rustica*) and Cliff swallow (*Hirundo pyrrhonota*), which are non-colonial breeders.

Very few studies compared individuality between populations of different densities in a single species. Previous work on Cape fur seals found that the individuality in two call-types involved in parent-offspring communication was higher in the larger colony^26^. The authors also found high individuality in the territorial calls of male Cape fur seals; however, they did not have data to compare individuality in territorial calls in both populations. They use the index of vocal stereotypy (IVS) as an individuality metric in their study which corrects DS by chance levels (IVS = DS/chance assignment; chance assignment = 100/number of individuals). This metric has the same issue as DS, as also discussed by the authors, i.e. it depends heavily on the number of individuals in the sample. Recalculating our values indicates that the IVS values and differences between studied conditions in our study would be comparable if not exceeding those found by Martin et al.^26^, (Fig. 3) for a comparable number of individuals (IVS HIGH = 19.29; IVS LOW = 17.47; IVS CLUMPED = 12.25; IVS ISOLATED = 7.89).

To our knowledge, our study is the first work to provide some evidence that high local density of conspecifics (CLUMPED vs. ISOLATED males) is associated with an increase of vocal individuality in territorial calls of a monogamous, pair-living species. Our results contradict two previous studies on the same topic. Blumstein et al.^35^ studied individuality in the songs of seven species of passerines and did not find any relationship between population density and the degree of individuality (Beecher’s statistic) in their songs. This study analysed features of entire songs (e.g. song duration, song rate, minimum and maximum frequency of song, number of syllables, syllable rate, features of different syllables and notes, etc.) coming from different species with quite varied vocalisations (different set of features for different species). However, songbirds may encode individuality specifically in certain parts of their songs^100,101^. Therefore, the analysis might be confounded by including song parts with different signalling functions^102,103^. In the second study, Delgado et al.^36^ suggested that high population density may even reduce vocal individuality in Eurasian eagle-owls (*Bubo bubo*). Here, they used vocalisations from a single population only and calculated discrimination scores from Discriminant Function Analyses (DFA) based on four spectral (minimum and maximum frequency, bandwidth and dominant frequency) and four temporal variables (total duration of call and durations of call sections with rising, stable and decreasing frequency). They then compared their results with those of a different study^104^. However, the results of both studies may not be directly comparable (slightly different sets of extracted acoustic features, different methods of feature extraction, different spectrogram settings, different sample sizes, etc.). Actually, both studies indicate very high vocal individuality with the difference in DS smaller than in our study (Delgado et al., DS = 95.8%; Lengagne et al., DS = 100%). Our study overcomes both these previous shortcomings by focusing on simple calls that function in individual recognition^61^, and by direct comparison between populations and within a population using the same analysis methods.

In territorial and largely sedentary species such as owls, individuals can benefit from having highly distinctive calls. Individually distinct vocalizations can allow neighbours to recognize one another and thus reduce unnecessary aggressive encounters, saving both time and energy^34^. Little owls, for example, are able to discriminate between neighbour and stranger calls^61^. Given that many owl species encode strong identity signatures in their calls^43^, such recognition could be particularly advantageous in minimizing misdirected aggression towards strangers, especially when the social environment becomes demanding.

### Study limitations

Our study does not come without limitations. We realise that comparing two populations can lead to false generalisations, because differences in vocal signals between two populations can arise due to many reasons besides the population density^105^. Ideally, we would need a sufficient number of replicates from other HIGH-LOW density and CLUMPED-ISOLATED conditions. On the other hand, this is a problem of many other pioneering studies^106^ and meta-analyses can be run on studies like this when their number accumulates in future. We believe our results are highly useful for better planning future studies and meta-analyses. While we could not include more replicates, we used two different approaches (HIGH-LOW density, CLUMPED-ISOLATED males) and came to very similar results in both cases.

Further, higher vocal individuality in the HIGH-density population was indicated by higher discrimination score and Beecher’s information statistic, which are both population-wide metrics. However, this pattern was not confirmed by significant differences in individual acoustic niche overlaps like in the case of comparison between CLUMPED and ISOLATED males. One reason possibly hindering the assumed difference in acoustic niche overlaps between populations might be the spatial distribution of males in both populations. Both HIGH and LOW populations indeed included males with and without immediate neighbours which could diminish possible differences between the populations if local spatial distribution is the decisive factor. Our study suggests that focusing on the immediate social environment is probably more promising for future studies rather than large-scale comparisons of overall population density patterns. In some species living in crowded social conditions, vocal identity becomes more pronounced in chicks at the time of fledging, i.e. with increased exposure and interactions with conspecifics^107–109^. Our results are similar to those found in some other studies on vocal interactions (e.g. ^66,95^). In Yellowhammers (*Emberiza citrinella*), differences in individually specific rhythms, for example, are largest between the closest neighbours^110^. The importance of immediate social environment might be especially important in the case of sedentary birds such as little owls.

Another explanation could lie in the fact that recordings were collected by different methods for both analyses. For the first comparison between populations, recordings were made after playback provocation, while recordings of spontaneous calls were used to compare individuality in CLUMPED and ISOLATED males within the high-density population. The use of playback has been shown to have effects on vocal responses of different species including changes in vocal output, timing, frequency and content^111–115^. Therefore, use of playback, in general, might be assumingly associated with increased within-individual variation of calls eventually masking differences in acoustic niche overlap between the HIGH and LOW conditions. On the other hand, studies on owls successfully use playback elicited calls to identify individuals within and across breeding season, suggesting little effect of playback on individually distinct call features^116–119^. Separate analyses of between- and within-individual variation in call features in playback elicited and provoked calls might inform us about how individual distinctiveness is maintained under different conditions. We cannot provide direct assessment of effects of playback on vocal individuality as we currently do not have enough good quality recordings for a proper analysis. However, consistent PFC patterns can be found in spontaneous and provoked recordings of the same males (Supplementary information, Figure S1). Yet, we also observed that PFCs of individuals can change during and across years in both playback-provoked as well as in spontaneous calls, suggesting that PFC can be consistent or substantially modified in both spontaneous as well as in provoked recordings (PL, unpublished results).

Another limitation is the fact that we could not validate the identity of males in the case of spontaneous calls recorded using autonomous recorders. Despite our efforts to select calls of a single closest focal male (see Methods), we cannot exclude the possibility that neighbours of CLUMPED focal males might also be recorded and misidentified as the focal males. However, if multiple males were in fact wrongly pooled together as one, then this would lead to systematic bias and lower overall individuality in CLUMPED males. Our results, on the contrary, show that individuality is higher in CLUMPED males.

### Potential mechanisms behind high individuality in CLUMPED males

Various mechanisms exist that could be responsible for the higher vocal individuality in CLUMPED males compared to males living on isolated territories. For example, neighbours with dissimilar calls might settle next to each other leading to the establishment of vocally diverse clusters. Alternatively, sexual selection for high consistency of calls observed in some species^120,121^] might accidentally result in higher individuality. However, we did not observe a significant decrease in within-individual variation in our study that should be associated with higher consistency of calling. Yet another possibility could be that individuals in urban habitats (most of our CLUMPED males came from villages and ISOLATED males from rural areas) might be more diverse genetically, and consequently acoustically. However, a review on this topic indicates rather lower genetic diversity in urban populations^122^. In following studies, genetic samples might allow us to take individual relationships and genetic diversity of populations into account to rule out the possibility that higher vocal individuality is merely a consequence of larger genetic divergence within a population/sub-population. Also, high population density associated with urbanisation^123^ could result in not only signal divergence between urban and rural populations^124,125^, but also in increased complexity in the signals of urban-living individuals^126^.

Our study might also represent an example of some form of vocal plasticity in owls. Indeed, calling of little owls seems quite plastic (e.g. call sequences with gradual transitions between the call types^48^, but owls are typically considered to be vocal non-learners^127,128^. Simple forms of vocal plasticity include short-time modifications of call amplitude, pitch and timing in response to noisy conditions or conspecific calls; these are taxonomically widespread^129–131^ but have rarely been described in owls.

Individuals could also have several variants of calls in their repertoire and might use a dissimilar call in interactions with their neighbours to enhance individual recognition among local neighbours - a form of a contextual vocal learning^132^. At the extreme, our study might represent an example of innovation learning^132^ where calls of animals diverge from the models. Observations documenting innovation learning are extensive in vocal learners^133–136^ but remains relatively understudied in non-oscine species. However, there is some evidence for it, for example, in common loon males (*Gavia immer*), who changed the structure of their yodels to sound more different than those of the previous territory owner^137^, and in black-headed gulls (*Larus ridibundus*), where chicks raised in captivity developed calls deviating from conspecific call models^138^. While there is some evidence that bird clades previously thought to be vocal non-learners actually possess abilities that fall squarely within the boundaries of what constitutes vocal production-learning^128^, fascinating speculations about any form of vocal learning in owls would need to be confirmed by additional research.

## Conclusion

In this study, we show that vocal individuality in the territorial calls of little owls is influenced by their social environment, particularly, by the local conspecific density (CLUMPED vs. ISOLATED males). Similar results were previously reported only for group-living and colonial species. While qualitatively similar patterns were found at both spatial scales, our results indicate that distribution within local neighbourhoods might be more important (vocal individuality significantly higher in CLUMPED vs. ISOLATED males) than larger-scale population characteristics (non-significant trend for higher vocal individuality in HIGH vs. LOW average population density). Limitations of our study do not allow us to conclusively decide about the mechanism leading to patterns observed here. In the future, data is needed to also take into account the spatial and genetic relationships between individuals. Further, more longitudinal studies would be needed to document the development of individually distinct calls in owls, and their plasticity later in life. While little owls do discriminate between the calls of neighbours and strangers, it would be worth investigating how sensitive little owls, and other species in general, are towards variations in within- and between-individual call variation to confirm that acoustic patterns revealed in this study are ecologically relevant.

## Supplementary Information

Below is the link to the electronic supplementary material.

Supplementary Material 1

Supplementary Material 2

Supplementary Material 3

## Data Availability

Data available on figshare: [doi.org/10.6084/m9.figshare.29958992](http:/doi.org/10.6084/m9.figshare.29958992).
