# Supplementary material for: Social environment affects vocal individuality in a non-learning species
Source: Sci Rep. 2025 Dec 15;16:51. doi: 10.1038/s41598-025-29387-3 (PMC12765009; doi:10.1038/s41598-025-29387-3)
Supplement: Supplementary file 1 — Supplementary Material 1 [file 41598_2025_29387_MOESM1_ESM.pdf]

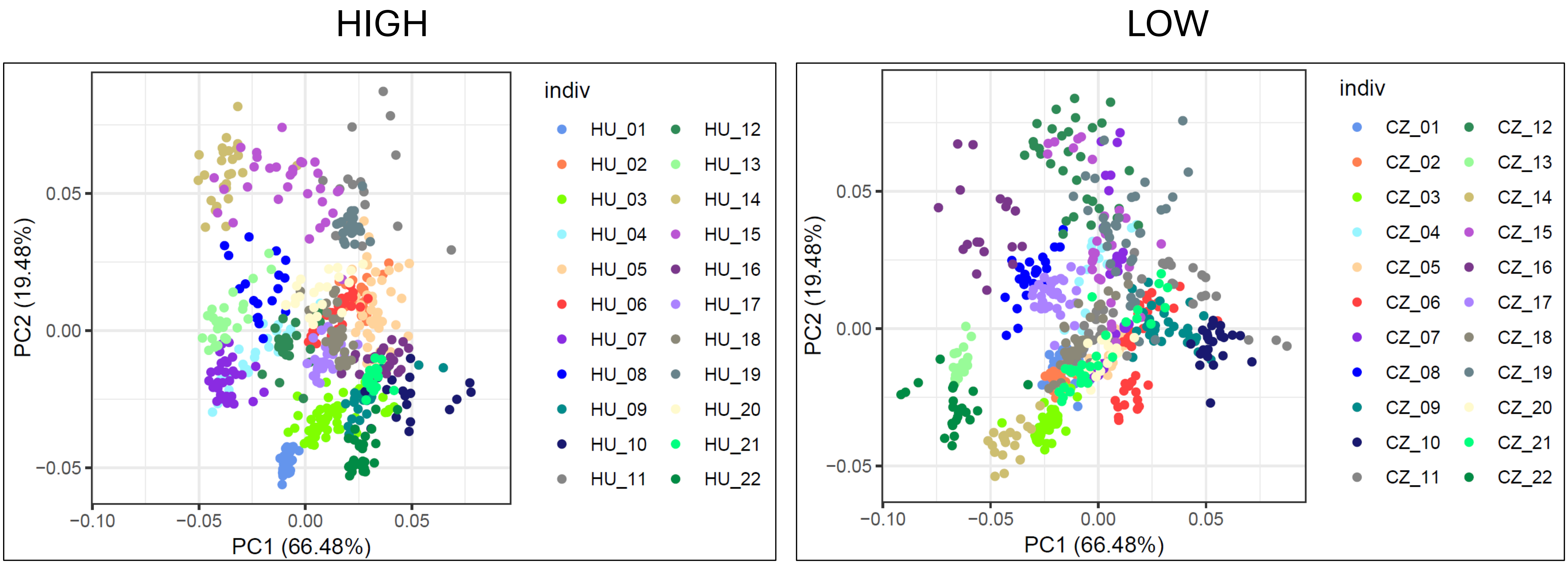

Fig. S2. Plots from a Principal Components Analysis done on call components (10 peak-frequency points and duration) for males from both HIGH and LOW density conditions.

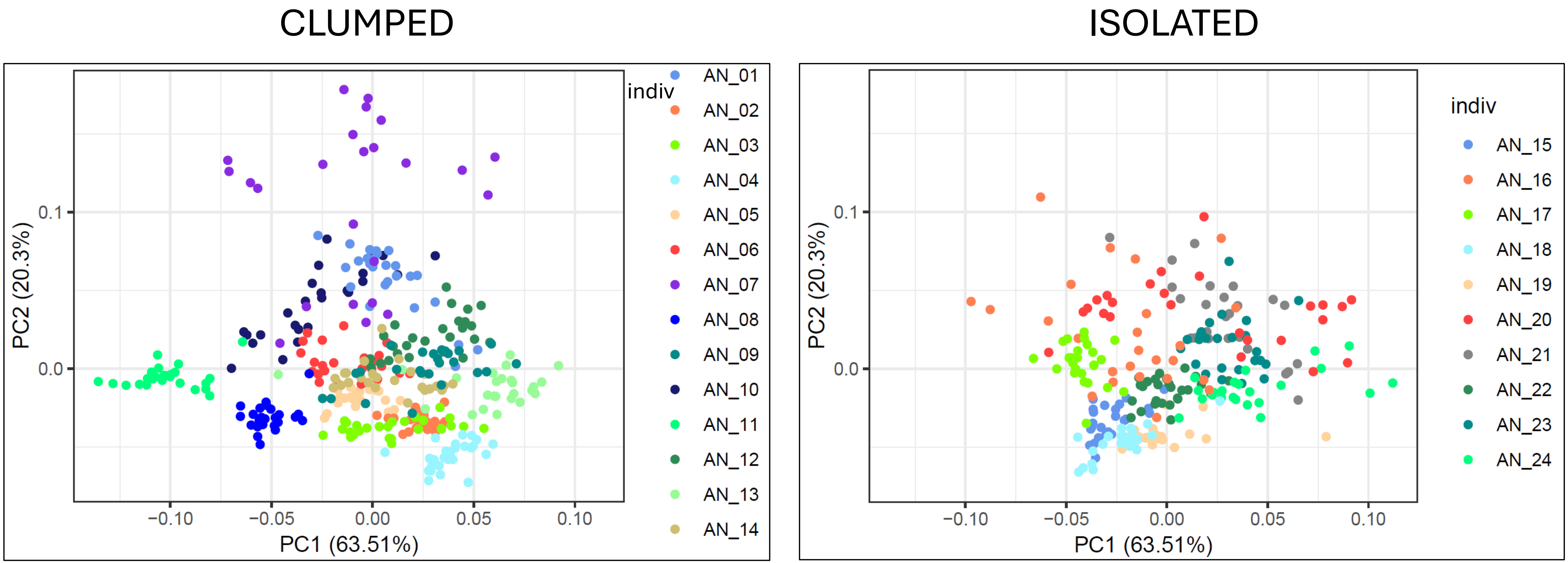

Fig. S3. Plots from a Principal Components Analysis done on call components (10 peak-frequency points and duration) for both CLUMPED and ISOLATED males.
