# Supplementary material for: Social environment affects vocal individuality in a non-learning species
Source: Sci Rep. 2025 Dec 15;16:51. doi: 10.1038/s41598-025-29387-3 (PMC12765009; doi:10.1038/s41598-025-29387-3)
Supplement: Supplementary file 2 — Supplementary Material 2 [file 41598_2025_29387_MOESM2_ESM.docx]

**Supplemenatry text, S4**

**Survey protocol for Little Owl distribution in the study sites**

In both our study sites, the initial surveys to estimate little owl (*Athene noctua*) population density were conducted using tape recorded stimulation of the male territorial call, which is the most widespread accepted method to confirm the little owl’s presence (see Van Nieuwenhuyse et al., 2023), and has been applied successfully to monitor this species (Šálek & Schröpfer, 2008; Šálek et al., 2013; Clewley et al., 2016; Chrenková et al., 2017). Following Johnson et al. (2009), a two-minute-long sequence of little owl territorial calls was broadcasted and repeated three times, with one-minute-long pauses in between repetitions. Playback was stopped immediately upon hearing the response of an individual. Surveys focused mainly on human settlements - such as agricultural buildings, old or abandoned buildings, residential areas, and settlement edges - where the species is commonly expected (Šálek & Schrӧpfer 2008; Dobrý, 2011; Šálek et al. 2013, 2016), but also included semi-natural habitats like parks, gardens, orchards, or pollard willow stands). Fieldwork took place in March and April of 2013 and 2014, which overlapped with the courtship period, when little owl males most actively produce territorial hoots in Central Europe (Exo 1988). Monitoring was carried out from sunset and continued throughout the night. A site was considered occupied if an owl was detected at least once during the survey period.

1. Chrenková, M., Dobrý, M. & Šálek, M. Further evidence of large-scale population decline and range contraction of the little owl Athene noctua in Central Europe. *Folia Zool* **66**, 106–16 (2017).
2. Clewley, G. D., Norfolk, D. L., Leech, D. I., & Balmer, D. E. (2016). Playback survey trial for the little owl Athene noctua in the UK. *Bird Study*, *63*(2), 268-272.
3. Dobrý, M. (2011). The abundance of the little owl (Athene noctua) in Podunajská rovina lowland in 2009 and 2010. *Slovak Raptor Journal*, *5*, 121.
4. Exo, K.-M. Annual cycle and ecological adaptions in the Little Owl (Athene noctua. *J Ornithol* **129**, 393–415 (1988).
5. Johnson, D. H., Van Nieuwenhuyse, D., & Génot, J. C. (2009). Survey protocol for the Little Owl Athene noctua. *Ardea*, *97*(4), 403-412.
6. Šálek, M. & Schröpfer, L. Population decline of the little owl (Athene noctua Scop.) in the Czech Republic. *Pol J Ecol* **56**, 527–534 (2008).
7. Šálek, M. *et al.* Scale-dependent habitat associations of a rapidly declining farmland predator, the Little Owl Athene noctua, in contrasting agricultural landscapes. *Agric Ecosyst Environ* **224**, 56–66 (2016).
8. Šálek, M., Chrenkova, M. & Kipson, M. High population density of little owl (Athene noctua) in hortobagy national park, Hungary, Central Europe. *Polish Journal of Ecology* **61**, 165–169 (2013).
9. Van Nieuwenhuyse, D., Van Harxen, R., Johnson, D. H. & De Raedt, J. *The Little Owl: Population Dynamics, Behavior and Management of <I>Athene Noctua</I>*. (Cambridge University Press, 2023).
