# Supplementary material for: Social environment affects vocal individuality in a non-learning species
Source: Sci Rep. 2025 Dec 15;16:51. doi: 10.1038/s41598-025-29387-3 (PMC12765009; doi:10.1038/s41598-025-29387-3)
Supplement: Supplementary file 3 — Supplementary Material 3 [file 41598_2025_29387_MOESM3_ESM.docx]

**A)**


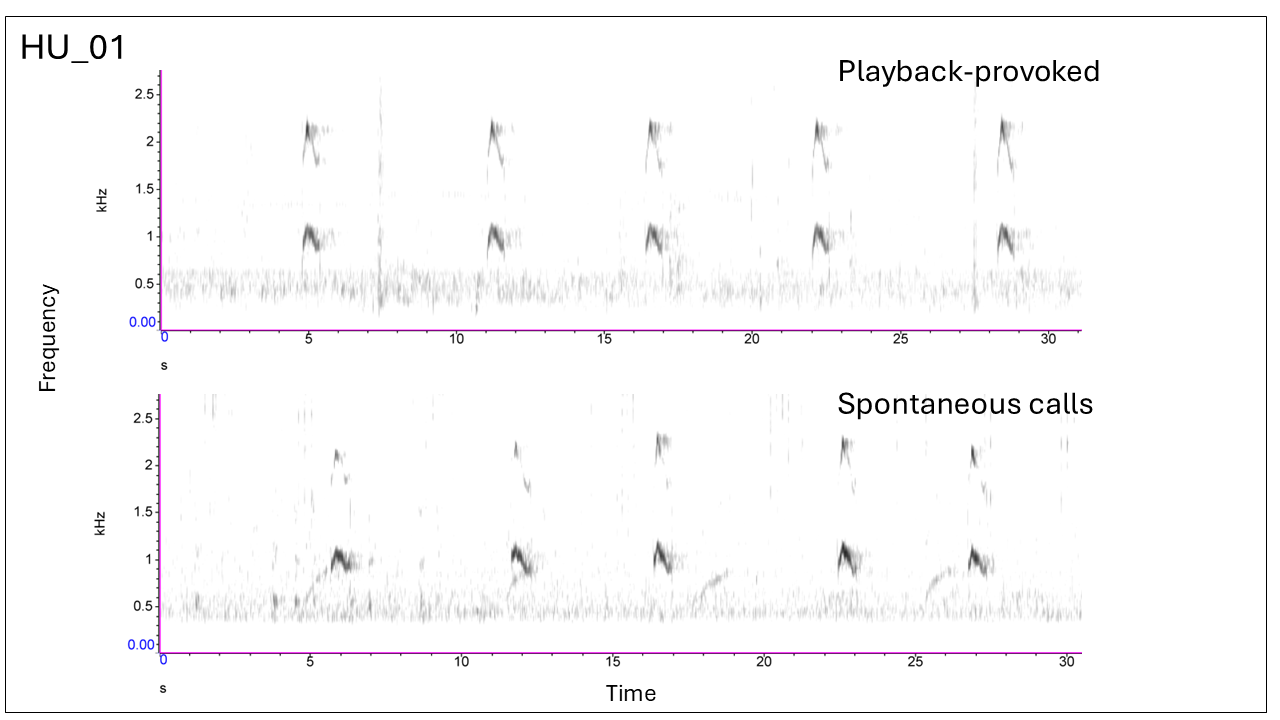


**B)**


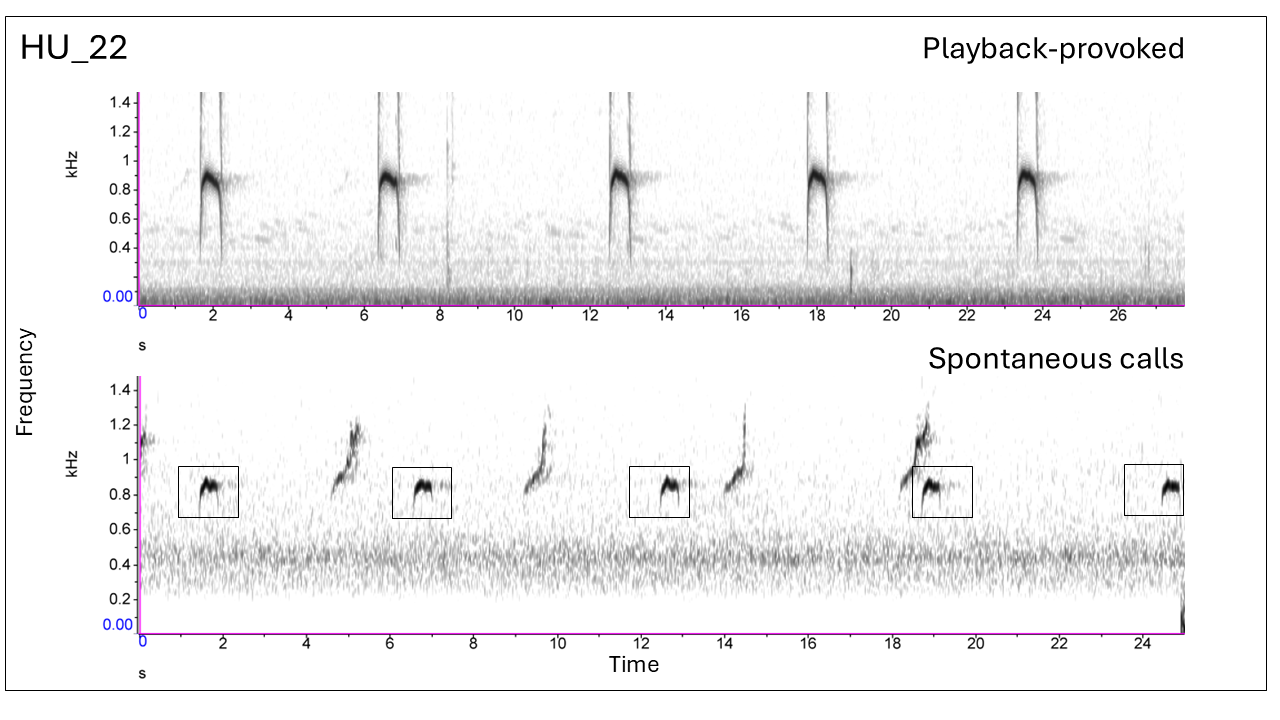


**Fig. S1.** A) and B) show spectrograms of calls recorded using playback provocation and spontaneous calls recorded using passive acoustic recorders, for individuals HU_01 and HU_22 respectively. In the lower spectrogram of panel B (Spontaneous calls), calls from two males are seen. We marked the calls from the focal male, i.e. the one seen in the upper spectrogram (Playback-provoked) of panel B, with black rectangles to show the similar call-pattern when recorded with both methods.
